# The expression of αA- and βB1-crystallin during normal development and regeneration, and proteomic analysis for the regenerating lens in *Xenopus laevis*

**Published:** 2011-03-23

**Authors:** Yongqing Zhao, Furong Ju, Yuanlin Zhao, Lei Wang, Zhenglong Sun, Mingxin Liu, Lan Gao

**Affiliations:** 1 School of Life Sciences, Lanzhou University, Lanzhou, Gansu, China; 2 Department of Chemistry, Tsinghua University, Bejing, China

## Abstract

**Purpose:**

To explore the expression of the lens crystallins **(**αA- and βB1-crystallin) in *Xenopus laevis* embryonic lens development and regeneration and to analyze the order of different crystallins generated in the regenerating lens.

**Methods:**

Real Time-PCR, Immunofluorescence, and 2D-PAGE were used to analyze the expressions of αA-crystallin and βB1-crystallin, and related factors during embryonic lens development and regeneration in *Xenopus laevis*.

**Results:**

αA-crystallin and βB1-crystallin were first detected at stage 29/30 during normal development, and the two crystallins were simultaneously detected in regeneration. During embryonic lens development, the relative expression level of the βB1-crystallin gene was higher than that of the αA-crystallin gene. In the process of the lens regeneration, however, the relative expression level of the βB1-crystallin gene was lower than that of the αA-crystallin gene. Throughout embryonic lens development, the two crystallin transcripts showed the same variation trends, and similar occurrence did in the regeneration process. Crystallins showed different localization and distribution during the ontogeny and regeneration, especially in the lens fiber region. 2D-electrophores revealed the patterns of the sequential synthesis of crystallins, with regard to the different classes and apparent variations of some auxiliary regulatory factors.

**Conclusions:**

The ontogeny and localization of the crystallins during embryonic lens development and regeneration indicated a different development program, although they have identical origins, the ectoderm. The expression level of crystallin transcripts displayed a consistent variation tendency, but the presence of appreciable differences was still exposed. In addition to stably producing the crystallins of different classes in accordance with established procedure, these auxiliary factors may perform the function, to some extent, because of significant changes in their expression throughout the process of lens regeneration.

## Introduction

Many organisms display a remarkable ability to replace missing or damaged tissues [1]. The focus of attention upon lens regeneration is largely because complete lentectomy in some members of one group of amphibians, namely, larval and adult urodeles, the newt, is followed by lens regeneration from the papillary margin of the dorsal iris. As we know, the newt is one of the few adult vertebrates that can regenerate the lens after damage or removal. Newt lens regeneration is characterized by the process of transdifferentiation, whereby terminally differentiated pigment epithelial cells of the dorsal iris dedifferentiate, proliferate, and then differentiate into lens cells [2,3]. As the urodeles amphibian, newt is much better at regeneration and can restore limbs, tails, retina of the eye and heart tissue, even as adults [4-6]. Among the other groups of amphibians, anuran, there are several species in which the lens does not regenerate [7]. However, *Xenopus laevis* is a unique anuran amphibian in terms of tissue source regeneration, which has the ability to regenerate a lens from the inner layer of the outer cornea [8,9], even in other members of the genus *Xenopus* [10,11]. After lentectomy, it can regenerate a new lens through the process of corner–lens transdifferentiation only in the larval stage [8]. The origin of the regenerated lens in *Xenopus laevis* is identical to that of the embryonic lens, which develops in normal ontogeny, because the inner layer of the outer cornea derives from the head surface epidermis. Interestingly, *Xenopus tropicalis* can also regenerate lens after the lens removal, but its success rate is much lower than that in *X. laevis* [11]. The regeneration of the response to injure occurs rarely and in a limited way among the well characterized vertebrate model organisms. Mice can regenerate their extreme digit tips and zebrafish can also regenerate their fins, brain, and heart tissue. The events of lens regeneration are found and have been studied extensively in rabbits, and have been extended to mice [12-14].

*Xenopus laevis* is probably the most well studied anuran amphibian in laboratories. In the developmental biology field, it is often used as the model species. Many genes in *X .laevis* have been identified, and a wide variety of molecular biology techniques has already been established for this species. Like other vertebrates, lenses express high levels of proteins as crystallins. An important feature of the lens is that it continually grows throughout life and accumulates cells in its outer layer without any protein turnover. Because of this feature and the pattern of cell accrual, it is an ideal tissue to study from a normal growth and from induced regeneration. Changes in lenticular protein distribution are a result of changing patterns of synthesis, especially in the two processes. Crystallins are major structural proteins in the lens. There are the three major classes: α-,β-, and γ-crystallins. The β- and γ-crystallin polypeptides are members of a related βγ-crystallin superfamily [15]. The accumulation of different crystallins is temporally and spatially regulated in the lens during development, making crystallins useful for investigating differential gene expression during cellular differentiation. Expression of these major crystallins during the embryonic lens development in *Xenopus laevis* was previously studied by immunohistochemistry and in situ hybridization [16-18]. In previous studies, the antiserum against total lens proteins gave rise to signals in both lens fibers and lens epithelium. Between lens regeneration and embryonic lens development in *Xenopus laevis*, the reported data indicated some similarities [17,19-21], but it also proved the existence of slight differences [22,23].

Once the original lens is removed, cells of the inner layer of the cornea epithelium begin to value-add and thicken as a placode to ultimately form a lens vesicle that differentiates primary and secondary fiber cells that contain lens crystallin proteins. This process is triggered by factors produced by the neural retina [23]. After lens is removed, injured tissues would produce inflammation. Previous studies suggest that the process of inflammation can promote regeneration in other systems [24]. Not only that, but also the process of inflammation associated with injury of the lens promotes axonal regeneration in the optic nerve [25]. Some researchers have proposed that the development of immune specificity and systems that promote inflammation, tissue repair may contribute to the loss of regenerative capacity in most vertebrates [26-29]. However, recent discoveries have been reported successful lens regeneration in adult frogs after metamorphosis, implying that after frogs complete metamorphosis, regenerative ability is recovered to some extent [30].

The widespread occurrence of regeneration among the Metazoan indicates that regeneration represents an ancient condition of metazoan biology [26,29]. Some studies have been done by comparing gene expression in *Xenopus* lens regeneration with gene expression in other regeneration system, for the purpose of being core molecular components in widespread occurrence of regeneration. Many transcription factors play important roles in the eye development, including paired box 6 (Pax6), prospero homeobox 1 (Prox1), avian musculoaponeurotic fibrosarcoma (MAF) protooncogene (Mafs), sex determing region Y–box 3 (Sox3), sine oculis homeobox 2 (Six2), orthodenticle homeobox 2 (Otx2), etc. The researchers have proved that the formation of the lenses require Otx2 in mice [31]. Sox3 also plays an important role in eye development and sox proteins are involved in regulating crystallin expression [32-34]. Prox1 and Mafs are well known that they are essential for lens fiber cells differentiation and can regulate the expression of crystallins [35,36]. Indeed, pax6 is involved in lens cell differentiation and crystallin gene expression, and is a master regulator of eye development [36,37]. Studies have revealed a relatively small subset of genes with overlapping expression by comparing gene expression in the two processes [38]. Seven hundred thirty-four unique genes were identified from a subtracted cDNA, which was prepared during the early development of lens regeneration in *Xenopus laevis* [38,39]. Some of the identified genes are transcription factors and cell signaling factors, and a considerable portion represent unknown transcripts. In addition, it is proposed that the processes of embryonic lens development and lens regeneration are closely related [40-42]. At the same time, Malloch et al. [38] lent further support to the view because some genes are expressed in lens regeneration, also expressed in normal development, including some of the genes mentioned above.

As *Xenopus laevis* development varies according to rearing conditions, these stages (Freeman described five distinct regeneration stages) should be a comparison of the results generated by different researchers.

To study whether there were differences in the distribution and sequential synthesis of lens proteins during the two processes, the study analyzed the spatio-temporal expression of αA-crystallin and βB1-crystallin from ontogeny and localization. Meanwhile, components of regenerated lenses were examined and some auxiliary regulatory factors were analyzed by 2D-MS.

## Methods

### Animal

*Xenopus laevis* embryos were obtained by hormone induced mating, kept at a temperature of 20 °C, and staged according to the normal table of Nieuwkoop and Faber [43]. In preparation for surgery, tadpoles were anaesthetized to remove the lenses.

### Crystallin gene clones and protein expression

Total RNA was isolated from the *Xenopus laevis* lenses collected from stage 50 to 55 tadpoles, and then was reversed transcribed into cDNA. The entire open reading frame of a cDNA encoding the full-length *X. laevis* αA-crystallin and βB1-crystallin was amplified by means of polymerase chain reaction (PCR). Nco I and Psc I restriction endonuclease sites were created upstream of the start codon using the primer 5′-CCA TGG ATA TCA CCA TTC AGC ACC-3′ and 5′-ACA TGT CTC ACA CAT CCA AAC C-3′, respectively, while at the same time, a Hind III restriction endonuclease site was created downstream of the translational stop codon using the primer 5′-AAG CTT GGA GGA TGA GCC TGA TTT CTC-3′ and 5′-AAG CTT CTT GGT TGT TGC AAT TAC-3′, respectively. The primers were synthesized from Invitrogen (Shanghai, China). The resulting 555 bp and 741 bp fragments were digested with Nco I and Hind III (Takara, Tokyo, Japan) at 37 °C for 5 h, cloned into pET28 expression vectors (Invitrogen) digested by Nco I and Hind III (Nco I and Psc I are isocaudarners). These two recombinants were verified by DNA sequencing. *Xenopus laevis* recombinant αA- and βB1-crystallin proteins were expressed in Rosetta (DE3) cells and purified by a nickel affinity column.

### Preparation of antisera against *Xenopus laevis* αA-crystallin and βB1-crystallin

A concentration of purified fusion proteins was examined by Bradford. First, 50 μg of recombinant αA-crystallin protein was mixed with 1 ml Freund's incomplete adjuvant, and then was injected into a Kunming mouse. Then 1 ml Freund’s incomplete adjuvant was replaced with 1 ml Freund’s complete adjuvant, which was mixed with antigen protein and injected into the mouse three times at weekly intervals. One mg of recombinant βB1-crystallin protein replaced αA-crystallin protein, and was injected into a rabbit by the same method. Finally, antisera were obtained and purified.

### First-strand cDNA synthesis and Real-time PCR

The study collected embryos at the normal developmental stage according to Nieuwkoop and Faber [43]. Total RNA was isolated from individual embryos using TRIzol (Invitrogen), according to the manufacturer’s instructions. To remove genomic DNA contamination, RNA was digested by RNase-free DNase I (Promega, Madison, WI) and then purified. Synthesis of first-strand cDNA was performed using reverse transcription reagents (Takara, Tokyo, Japan). Total RNA (1 μg) was dissolved in 13 μl solution containing 1 μl oligo (dT)18 and 12 μl RNase-free water. To denature the sample the solution was incubated at 70 °C for 10 min, and immediately cooled on ice for 2 min. Reverse transcription was performed by the addition of 4 μl 5× first strand synthesis buffer, 1 μl dNTP mixture (2.5 mM), 1 μl RNase inhibitor, and 1 μl M-MLV RTase and incubated at 37 °C for 1 h. The reaction was terminated by heating to 85 °C for 7 min. Finally, all samples were analyzed by real-time PCR.

Eyeballs of regenerating lenses were extracted, according to the time sequence of regeneration, with the same operation.

Real time PCR was performed using a Bio-rad iCycler (Bio-Rad, Hercules, CA). Samples were set up in 25 μl volumes containing 12.5 μl 2× premix Ex Taq (Takara), 0.5 μl Forward primer (10 μM), 0.5 μl Reverse primer (10 μM), 2.5 μl SYBR Green I, 2 μl template, and 7 μl sterilized distilled water. Reaction was performed under the following conditions: 95 °C for 3 min, followed by 45 cycles of 95 °C for 5 s, and 60 °C for 20 s. All reactions were performed in triplicate. Glyceraldehyde-3-phosphate dehydrogenase (*GAPDH*) was included in each assay as a loading control. Primers for real-time PCR are shown in Table 1.

**Table 1:** Primers used for real-time PCR.

**Gene**	**Accession number**	**Direction**	**Sequence**	**Product length (bp)**
αA-crystallin	D88185	Forward	5′-CAGGTCTTTGGTGAGGGAATG-3′	87
		Reverse	5′-GGAGAGGTTCTGCTTGTAGTAGGG-3′	
βB1-crystallin	D88186	Forward	5′-ATGTGGAAACCTTGGGGAAA-3′	104
		Reverse	5′-ACATCTCACCACGGAAGTTGG-3′	
*GAPDH*	BC043972	Forward	5′-AGCTGTGGAGAGATGGCAGAG-3′	139
		Reverse	5′-ACATCTCACCACGGAAGTTGG-3′	

The Table displays the specific primers of each gene, and appropriate product length ensures specific amplification.

### Immunofluorescence

Staged embryos and different lens regeneration-timed tadpoles were fixed in 4% PFA in PBS (PH 7.4) overnight at 4 °C. After dehydration through a series of graded ethanols (50%, 75%, 85%, 95%, and 100%) for 15 min each, samples were treated with xylene, and finally, embedded in paraffin wax. The tissues were cut into 6 μm sections. Paraffin sections were treated with xylene and ethanol, washed with PBS, and then repaired antigen. Slices were blocked with 10% goat serum. Polyclonal rabbit antiserum against *Xenopus laevis* βB1-crystallin was used at a dilution of 1:200; the same dilution was used for polyclonal mouse antiserum against *Xenopus laevis* αA-crystallin. Sections were incubated with the two antibodies at 4 °C overnight, washed with PBS three times, 5 min each, and then incubated with rhodamine conjugated secondary antibody (goat anti-rabbit IgG) and FITC conjugated secondary antibody (goat anti- mouse IgG, diluted 1:200) for 60 min at 37 °C. Various negative controls were performed. After a final wash, the slices were coverslipped and examined with a confocal microscope (Zeiss LSM 510; Carl Zeiss, Jena, Germany).

### Sample preparation for protein analysis —two dimensional electrophoresis

Using the microscope, eyeballs of regenerating lenses were collected from experimental groups at different times (3, 5, 7, 9, and 15 days after lentectomy) and control eyes were collected (0 day). The samples were pooled and ground to a power with liquid nitrogen. The powder was dissolved in lysis buffer that contained 7 M urea, 2 M thiourea, 4% w/v chaps, 70 mM DTT, and 0.3% v/v bio-lyte ampholyte, for pH 3–10, and 1 mM PMSF, 10 mM Tris, and 0.5 mM EDTA. After suspension for 8 h, they were centrifuged at 13,523× g at 4 °C for 40 min. The supernatant was collected and precipitated with TCA-Acetone at the ratio of 1:7 at −20 °C for 6 h. After being spun at 18,407× g at 4 °C for 30 min, the supernatant was removed and the precipitate was washed with acetone 3 times, 5 min each. The precipitate was dried in air about 2 min and dissolved in lysis buffer as mentioned above for 4 h, and then was centrifuged at 37,565× g for 1 h at 4 °C. Protein concentration was determined by the Bradford assay and stored at −70 °C until further use.

### Two-dimensioned electrophoresis

Two-dimensioned electrophoresis was performed as follows: In the first dimension (isoelectric focusing) IPG strips (pH 3–10, 17 cm; Bio-Rad) were used according to the manufacturer’s instructions, and then SDS–PAGE was finished as the second dimension. Each IPG strip was loaded at 150 μg protein. After focusing, the strips were immediately equilibrated two times for 14 min each time. The equilibration solution contained 6 M urea, 2% SDS, 0.375 M (pH 8.8) Tris-HCl, and 20% glycerin. The DTT was added to the solution for the first equilibration; for the second, iodoacetamide replaced DTT. The second dimension was performed using 13% SDS–PAGE gel in the Protein II Device (Bio-Rad) for the separation. The electrophoresis was performed at a constant voltage (50 V) for 45 min and then changed to 200 V for 7 h, keeping the temperature at 16 °C. Afterwards, the gels were fixed for 6 h, and then washed with deionized water three times every five min. Finally, protein spots were captured and analyzed after the gels was stained with commasine brilliant blue G-250 (Sigma-Aldrich, Shanghai, China).

### Statistics

For real-time PCR, sample numbers of each sample group were 3 (n=3), and each sample contained 40 individuals (operation or no operation). For higher accuracy, each sample was performed in triplicate.

2D-electrophoresis analyses guaranteed reliable results for three parallel tests, and each sample group contained three samples (same operation).

## Results

**Figure 1:**
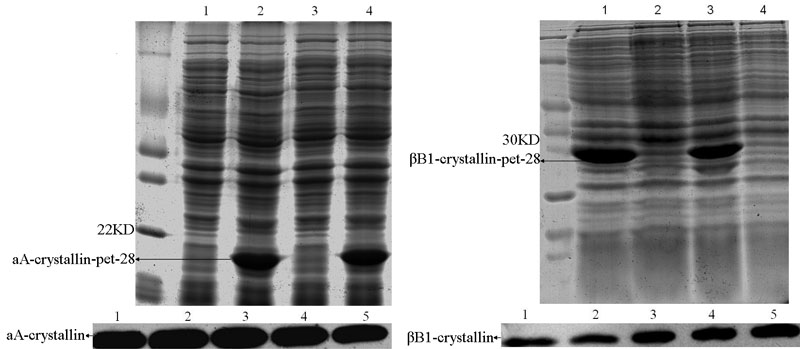
SDS–PAGE for induced expression of recombinant proteins (αA-crystallin-pet28, βB1-crystallin-pet28) and western blotting analysis for the two specific antibodies. Recombinant proteins were expressed in *E. coli* Rosetta (DE3). Their respective molecular weigh are 19 kDa and 23 kDa. In western blotting analysis, sample 1, 2, and 3 are total lens proteins; samples 4 and 5 are purified fusion proteins. By results, good specificity is shown.

αA-crystallin and βB1-crystallin of *Xenopus laevis* were cloned and crystallins were expressed successfully in Rosetta (DE3). By SDS–PAGE, 19 kDa and 23 kDa protein bands were observed (Figure 1). His-tagged fusion proteins were purified by a nickel affinity column. They were used as antigens and injected into the mouse and rabbit. After antisera were obtained, western blotting showed that the two polyclonal antisera had good specificity against αA-crystallin and βB1-crystallin of *Xenopus laevis* lens protein (Figure 1).

### The mRNA expression of αA-crystallin and βB1-crystallin in *Xenopus laevis* lens embryonic development and lens regeneration

**Figure 2:**
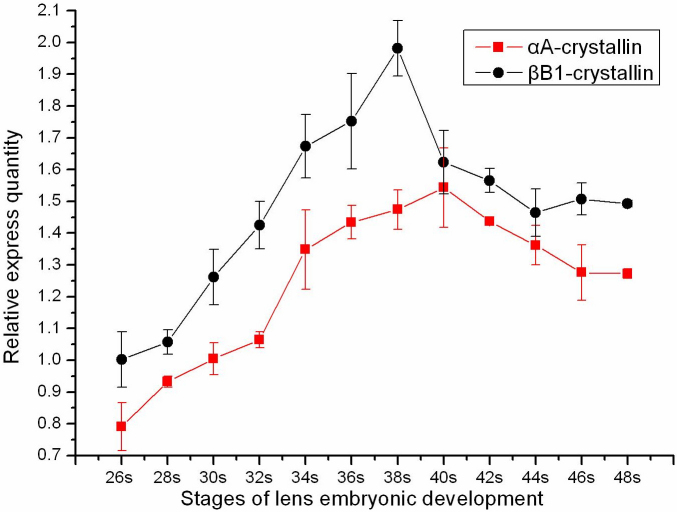
Real-time PCR analysis for transcripts of αA-crystallin and βB1-crystallin during the lens development. Stages are according to the normal table of Nieuwkoop & Faber. The red curve illustrates the relative expression of transcripts of αA-crystallin. From the beginning of expression at stage 26 to stage 38, it displayed an increasing trend. After stage 38, the expression began to decrease. Finally, the expression quantity maintained at a relative stable level. The same went for βB1-crystallin, which is shown by the black curve.

By Real Time PCR, the αA-crystallin signal and the βB1-crystallin signal were simultaneously detected at stage 26 (Figure 2). As lens development proceeded from stage 28 to 38, the mRNA expression of αA-crystallin and βB1-crystallin were gradually increased, at the same time. When primary and secondary lens fiber cells fully differentiated, their expression levels began to decrease after stage 38. As the lenses matured, expressions of these two crystalline genes were relatively stable. Throughout the developmental stages, they displayed the same variation tendency.

### Expression of crystallin genes in regenerating lenses of *Xenopus laevis*

**Figure 3:**
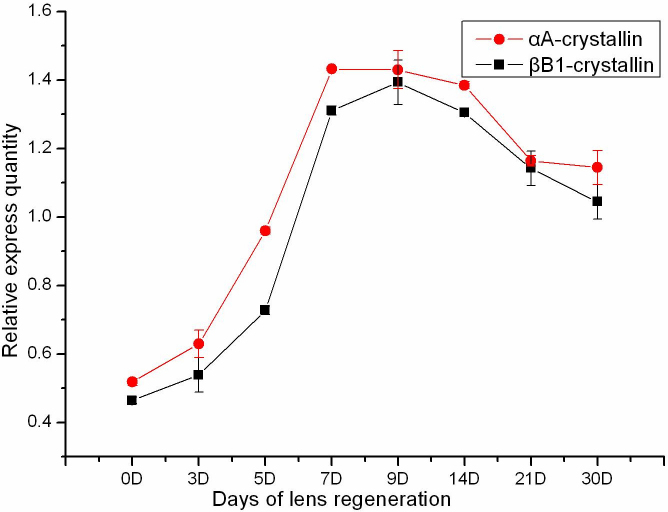
Real time PCR analysis for transcripts of αA-crystallin and βB1-crystallin during lens regeneration. The eyeballs of regenerated lenses were dissected at pre-operation, 0 day, 3 days, 5 days, 7 days, 9 days, 14 days, 21 days, and 30 days after lentectomy. The transcripts of two crystallins showed the same variation trends.

In these experiments, transcription variation of αA- and βB1-crystallin began 3 days after lentectomy (Figure 3). As the regenerating lenses developed, expression of the two crystallin genes displayed an increasing tendency. The expression quantity of αA-crystallin reached a peak on the 7th day, but the expression quantity of βB1-crystallin reached a peak on the 9th day. The expression quantity of αA-crystallin on day 9 and day 7 was almost the same. When regenerating lenses reached morphological maturation, expression of the two crystallin genes began to decline. Finally, the expression of αA-crystallin and βB1-crystallin maintained on a stable level with the regeneration process being finished. During the whole course of lens regeneration, the expression of the two crystallin genes also indicated the same variation tendency.

### Immunofluorescence of αA-crystallin and βB1-crystallin during embryonic lens development and regeneration

**Figure 4:**
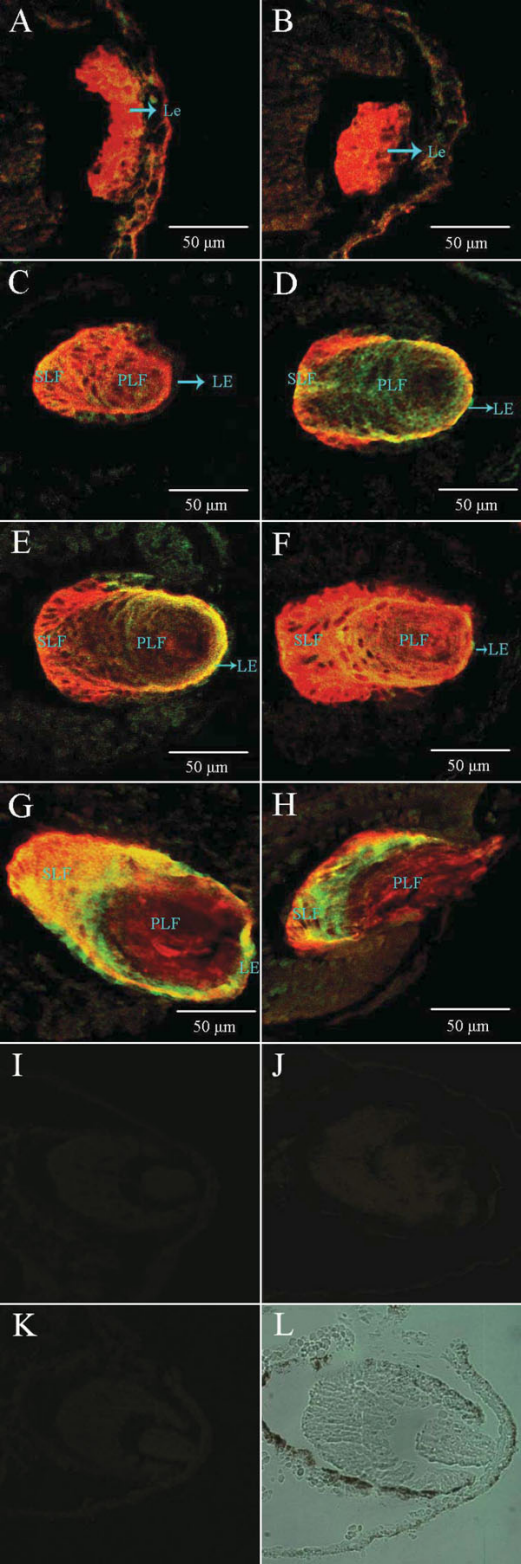
Immunofluorescence for αA-crystallin and βB1-crystallin during embryonic lens development. Sections double-stained with αA-crystallin and βB1-crystallin antisera at different developmental stages, analyzed by confocal microscopy. Some cells express predominantly βB1-crystallin (red) and some αA-crystallin (green). Overall, there is a strong co-localization of these two crystallin proteins throughout the lens cells. First positive immunofluorescence was detected at stage 29/30 (**A**). At stage 32 (**B**), a number of cells in the area of the lens rudiment where lens fibers will form. With further differentiation, the lens primary fibers and secondary primary fibers are formed during stage 34–46 (**C**-**H**). Negative controls: **I** (without antibodies); **J** (only secondary antibodies); **K** (only primary antibodies); **L**: differential interference contrast (DIC). Abbreviations: Le, lens; PLF, primary lens fiber; SLF, secondary lens fiber.

**Figure 5:**
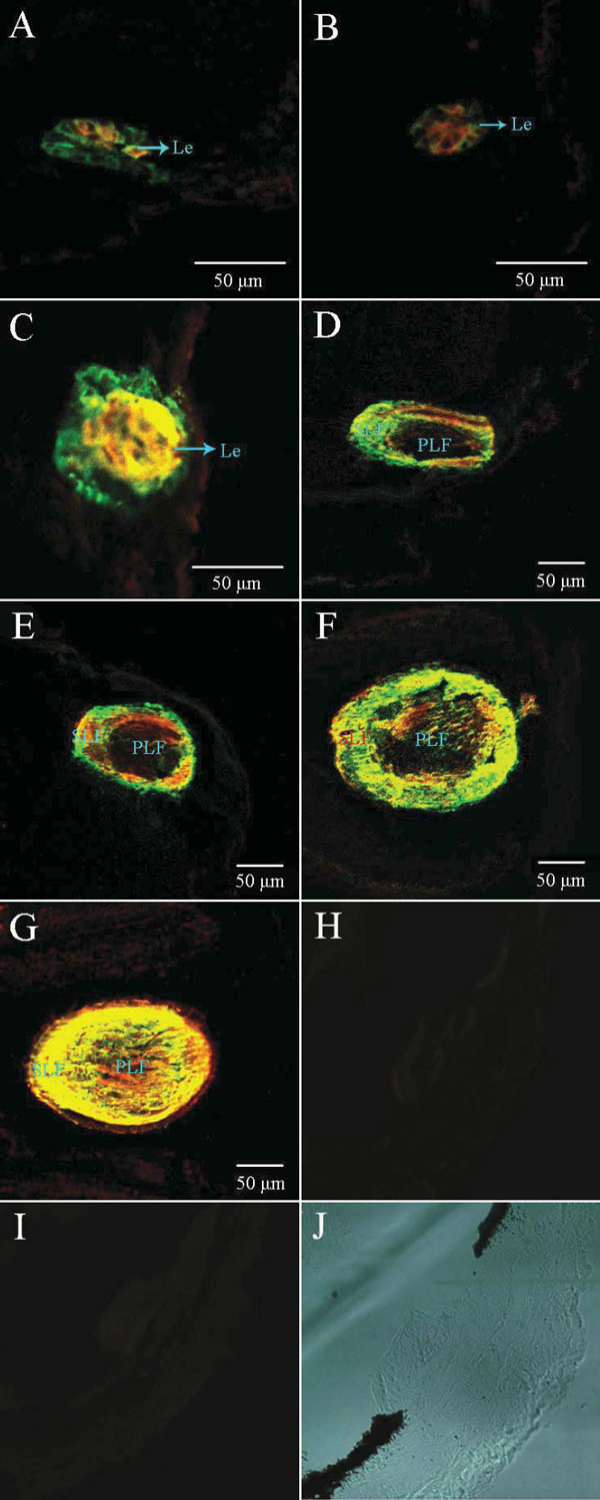
Immunofluorescence for αA-crystallin and βB1-crystallin during the lens regeneration. The images show the distribution of αA-crystallin and βB1-crystallin in the process of the regeneration, analyzed by confocal microscopy. The regenerated lenses were dissected at operation: 3 days, 5 days, 7 days, 9 days, 11 days, 15 days, and 21 days, as is shown in **A**-**G**, respectively. Negative controls: **H** (only primary antibodies); **I** (only secondary antibodies); **J**: differential interference contrast (DIC). Abbreviations: Le, lens; PLF, primary lens fiber; SLF, secondary lens fiber.

The prospective lens ectoderm was found to be negative for immunofluorescence. No immunofluorescence was detectable until the lens placode increased in thickness and changed in morphology (stage 29/30; Figure 4A), and signals (amplified signals) were captured. This observation indicated that these lens cells had been synthesizing a small amount of βB1-crystallin and less αA-crystallin. The same pattern of localization of immunofluorescence persisted at stage 32 (Figure 4B), and the irregular lens rudiment had become more clearly defined, at this stage, as a compact mass of centrally-located cells, surrounded by a peripheral, more loosely-arranged cell mass. With this initial inner mass of the lens rudiment differentiating into the lens fibers, more αA-crystallin was synthesized than during the early stage, and more loosely-organized cells became transformed into the external layer, and later, into the lens epithelium, where the two crystallins continued to have present and persistent expression. As lens development progressed (stage 34–46; Figure 4C-H), there was αA-crystallin to be expressed in the primary lens fibers, detectable by its immunofluorescence. With further differentiation, more and more expression of βB1-crystallin was displayed by the intensity of immunofluorescence. Finally, more βB1-crystallin was expressed than αA-crystallin in the primary fibers, but our data showed almost equal distribution of the two crystallins in the secondary fibers. During the regeneration process, no immunofluorescence was detected at day 0. Within one day after removal of the lens, the wound in the cornea had healed over. Immunofluorescence was detected on the third day after lentectomy (Figure 5A), and immunofluorescence was found at a loose clump of cells, which was formed from the inner layer of the outer cornea. By the fifth day of regeneration, a vesicle had been formed (Figure 5B), and immunofluorescence was detectable in the cells of the vesicle as well as in the cells formed on the third day. Data indicated that αA-crystallin and βB1-crystallin were co-located in the cells of the vesicle at day 5, and the expression level of βB1-crystallin was higher than on the third day as showed by the intense immunofluorescence. On regeneration day 7 (Figure 5C), regenerating lenses displayed morphological and structural change, increasing in size. More αA-crystallin and βB1-crystallin were obviously synthesized in the cells differentiating into primary fibers, detectable by the intense immunofluorescence. With further differentiation within the regeneration, the primary fibers became morphologically evident and the immunofluorescence in the vesicle was mainly located in the fibers. Regeneration occurred 11 days after lentectomy (Figure 5E), which was indicated by the development of differentiating secondary fibers and the growth of the lens. More αA-crystallin than βB1-crystallin was expressed in the region of the secondary fiber. However, βB1-crystallin prevailed over αA-crystallin in the primary fibers, from the observed results. At regeneration 15 days (Figure 5F), the two crystallins were equally distributed in the region of the secondary fibers, and almost the same pattern occurred in the primary fibers. Finally, the regenerated lens had fully matured morphologically on the 21st day (Figure 5G) and continued to be the same pattern of crystallin expression. It did not show any major structural change except the intensity of the immunofluorescence was increased throughout the area of the lens. Detectable immunofluorescence showed the ontogeny and localization of the two lens crystallins in *Xenopus laevis* lens regeneration.

### Proteomic analysis for the regenerating lens

**Figure 6:**
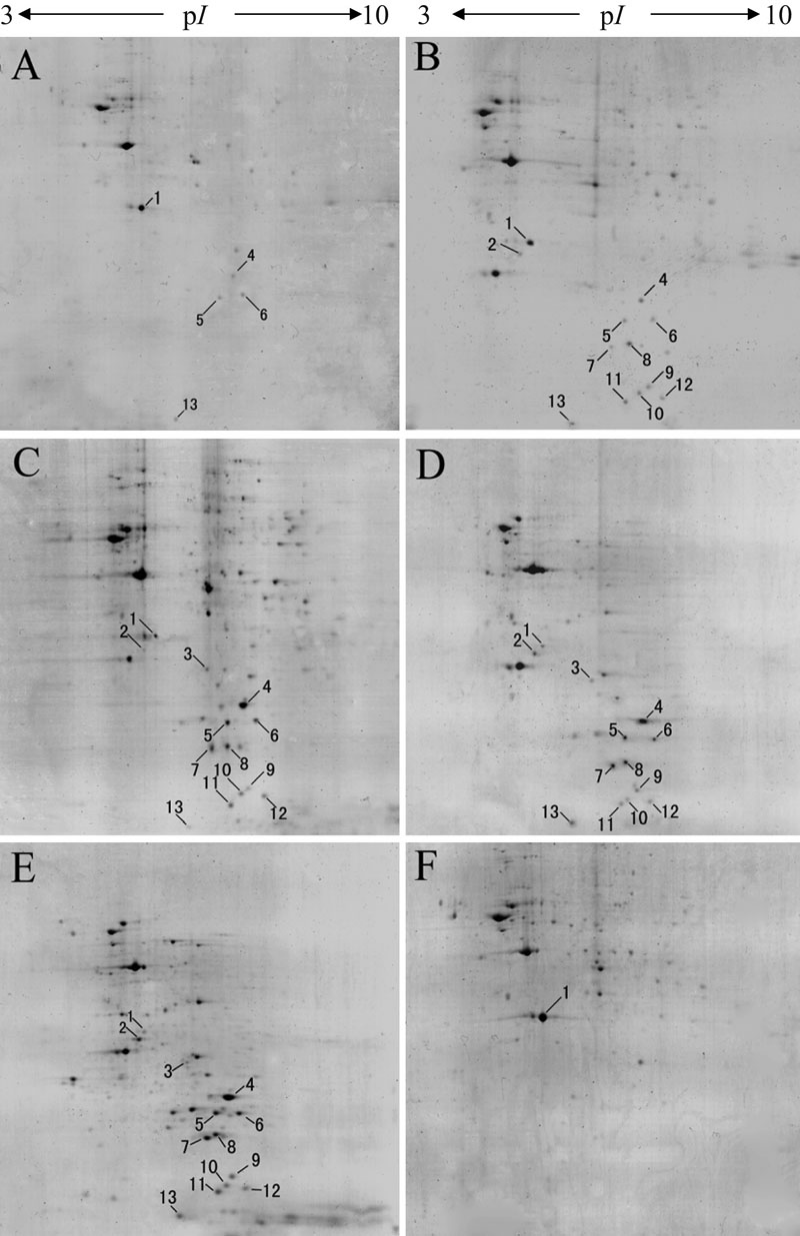
Two-dimensional electrophoresis photography of regenerated lens. **A**-**F**: The patterns of protein spots in regenerated lens (5 days, 7 days, 9 days, 11 days, 15 days, 0 day, respectively). The protein patterns are significantly different among all stages of regeneration. Although differences existed, some protein spots just showed the variation of expression quantity.

Proteomic analysis was performed to identify proteins that were expressed in the regenerating lens. The spots per gel were detected in the pH range 3–10 (Figure 6A-F), which was chosen for the analyses because of the apparent variation of the major protein population in the regenerating lens samples. The proteins were identified by MS (Table 2) including αA-crystallin, βB1-crystallin, βA2-crystallin, βA1-crystallin, retinaldehyde binding protein, centromere protein, guanine nucleotide-binding protein G subunit beta, and βγ-crystallin. There were fold changes in the expression of identified proteins (Table 3). The most significant results were that the crystallins were increasingly expressed and corresponding changes were produced in non-lens proteins. The lens regeneration appeared to produce different classes of lens proteins, which revealed that the pattern of the crystallins expression may be related with sequential synthesis. At the same time, the expression of some non-lens proteins varied from less to more, or more to less, with the start of lens regeneration. αA-crystallin expression was first detected at day 5 after lentectomy in the study, as observed in the case of βB1-crystallin on the same day. Not only the two crystallins, but also other crystallins presented from day 5 to day 15, and their expression gradually increased with the development of the regenerating lens.

**Table 2:** Proteins of regenerated lens identified by mass spectrometry.

**Spot number**	**Protein name**	**Access ID**	**Computed PI**
1	G protein subunit beta	gi|3023838	5.53
2	Retinaldehyde binding protein	gi|147903597	5.20
3	Centromere protein E	gi|147900710	6.10
4	βB1-crystallin	gi|147905564	6.82
5	βA2-crystallin	gi|148234150	6.32
6	βA2-crystallin	gi|148234150	6.32
7	β/γ crystallin (LOC494645 protein)	gi|52078358	6.23
8	βA1-crystallin	gi|32450481	6.39
9	Crygn protein	gi|138519900	6.24
10	β/γ crystallin (MGC84008 protein)	gi|49522149	6.52
11	Crygn protein	gi|138519900	6.24
12	β/γ crystallin (MGC84008 protein)	gi|49522149	6.52
13	αA-crystallin	gi|213623808	5.87

Identified protein spots can be divided into two types of protein: lens proteins and non-lens proteins. Proteins were produced in succession with the development of regenerated lens.

**Table 3:** Quantitative analysis of identified proteins.

**Spot number**	**0 Day**	**5 Days**	**7 Days**	**9 Days**	**11 Days**	**15 Days**	**Trends**
1	+	−4.825	−10.461	−35.919	−47.768	−115.991	-
2	0	0	+	1.017	37.396	54.159	+
3	0	0	+	5.897	7.729	9.906	+
4	0	+	2.790	38.229	60.061	111.573	+
5	0	+	−1.096	6.203	11.261	13.663	+
6	0	+	1.103	6.039	6.459	7.284	+
7	0	0	+	9.760	22.605	43.273	+
8	0	0	+	1.466	1.766	2.319	+
9	0	0	+	−2.868	4.284	11.995	+
10	0	0	+	2.549	−3.297	3.875	+
11	0	0	+	2.077	3.246	5.210	+
12	0	0	+	1.314	6.374	10.487	+
13	0	+	1.271	0.923	9.405	13.789	+

During lens regeneration, most proteins (lens proteins and some auxiliary regulatory factors) were gradually increasing with the further development of lens regeneration. When some proteins have not been shown in gels or expressed, especially in the early development of regeneration, their quantity could be denoted as “0” in our experiments. “+” represents that proteins have begun to be expressed.

## Discussion

There have been few attempts to elucidate the localization and time of appearance of the two important crystallins (αA-crystallin, βB1-crystallin) in *Xenopus laevis* embryonic lens development and regeneration. The comparison of molecular events, which take place in lens development in ontogeny and in regeneration in terms of the expression of crystallin genes and crystallin proteins, is the core subject of this study.

In this study, positive signals for the expression of αA- and βB1-crystallin mRNA were simultaneously detected, first at same stage (26), before the formation of lens rudiment. Significant increases occurred at later stages, with lens fiber differentiation and development. The rapid increase of the two crystalline genes demonstrated that lens cells were being formed and induced by the prospective lens cells. More transcripts of the two crystallins were expressed at stage 38 than during the early stage. After stage 38, the expression levels gradually decreased. The appearance of variation may be due to the disappearance of nuclei in the fiber cells, with the differentiating of fibers. Although decreased, expression of the two crystallin genes maintained a relatively stable level, which was needed for the ability of keeping synthesized structural proteins. Throughout the process, the expression levels of αA- and βB1-crystallin mRNA indicated the same variation trends, and the relative expression of βB1-crystallin was consistently higher than αA-crystallin. In the course of lens regeneration, the two crystallin genes showed the same variation trends, too. However, the relative expression of αA-crystallin was consistently higher than βB1-crystallin. Therefore, there were some differences during the two processes, as have been observed with respect to crystallin transcripts in previous studies [22,38,44]. αA- and βB1-crystallin transcripts were first detected in presumptive lens fiber cells of the regenerated lens vesicle, and subsequently, only in differentiated lens fiber cells at later stages. γ-Crystallin transcripts were not detected until early Freeman stage 4 of the regeneration and only in lens fiber cells. However, during normal development, αA-, βB1- and γ-crystallin transcripts were detected simultaneously in the lens placode and only in differentiated lens fiber cells at later stages of development. In contrast, recent reports demonstrated that the expression of βB1-crystallin during lens regeneration required the same promoter elements as those required during embryonic lens development, suggesting that elements of a shared regulatory network appeared to be operating in both of these lens-forming processes [41]. Interestingly, the lower expression levels of the two crystalline genes were detected in the current experimental groups of 0 day. This indicated trace expression in the non-lens tissue, which might be necessary for the transdifferentiation and the initiation of regeneration.

This study showed the ontogeny and localization of the two crystallin proteins during embryonic lens development and regeneration. The αA-crystallin and βB1-crystallin were first detected, simultaneously, at stage 29/30, which was different from the previous studies. With lens development and lens fibers differentiation, αA-crystallin and βB1-crystallin were both expressed in the secondary fibers, almost uniformly. In the primary fibers, βB1-crystallin was dominant, and preferred to αA-crystallin. However, the two crystallin proteins were simultaneously detected during the regeneration, which was consistent with normal lens development. In early regeneration, the external layers of regenerating rudiment indicated more expression of αA-crystallin. With further differentiation, the two crystallin proteins were co-located in fibers region, and almost homogeneously distributed in the primary fibers and the secondary fibers. From these results, differences existed during the two processes. Among the vertebrates, either a normally developing lens or a regenerating lens passes through a typical vesicle stage where the external cell layer that will give rise to the lens epithelium can be distinguished from internal cell layer that will develop into primary fibers. In *Xenopus laevis,* this vesicle is short lived, both in normal lens development and in regeneration. There were also other differences between the embryonic lens development and regeneration. In the latter, lens vesicles appeared much earlier and epithelium showed immunofluorescence of the crystallin earlier than in embryonic lens development. Although the two crystallins were co-located in many regions of the lens, there were differences in the distribution patterns in some regions, especially in the beginning of the lens development and regeneration. In newts, despite different origins of the lens in normal lens development and regeneration, the expression pattern of the two crystallin genes was similar in the two processes. It is noteworthy that the order of activation of the crystallin genes resembles embryonic lens development in newts more than in *Xenopus laevis* [45] because the γ-crystallin gene is delayed, relative to αA- and βB1-crystallin, but these crystallin transcripts were already expressed in the lens placode [22]. These findings indicate that there were some differences in the regulation of crystallins expression during regeneration versus development of the lens, as the transcription of crystallin genes has been examined in the process of lens regeneration [22,38]. Although the embryonic lens and the regenerated lens arise from the ectoderm, they exhibited different arrangements of genes and different procedures of protein distribution.

A perfect regenerated lens should have a healthy appearance and the histological arrangement of a new regenerated lens as well as an accurate protein composition. During the lens regeneration, αA-, βB1-, and βA2-crystallin were synthesized first, before other structural proteins, as shown in Figure 6 and Table 2. Other structural proteins were produced in turn, and accompanied by an increase of expression quantity with regenerated lens formation. αA-crystallin, for instance, which is an evolutionary relative of small heat-shock proteins [46], has been shown to act as a molecular chaperon and is able to convey thermotolerance [18,47,48]. Similarly, βB1-crystallin is a specific structural protein, as a sign of lens fiber differentiation [49]. The reason is probably that they are important for lens composition and development. Therefore, they are produced at the beginning of lens formation, and they accompany the whole development process. The sequential appearance may be necessary for the program.

It is known that, in lens regeneration, the inner layer of the outer cornea is dependent on inductive signals secreted from the neural retina, for initiation of lens formation [8,9]. It is also well known that pax6, prox1, Mafs, sox2, and others are important regulatory factors in the process of lens formation and development. However, the current study found that other factors might also be involved in lens regeneration. Retinaldehyde binding protein is the derivative of vitamin A, which can accelerate mitosis after lentectomy, and thus, enhance the dedifferentiation [50]. Centromere protein E is involved in cell division and proliferation. In lens regeneration, cells for stopping phase G_0_ are activated for the proliferation into phase G_1_. G-protein may perform a certain function when induced signals are transmitted after lens removal because it is a transmitter and can regulate the signals induced by hormone, neurotransmitter, and visual stimulation. Noelin-1 is a secreted glycoprotein and can promote the differentiation of the nerves, as reported in a previous study; perhaps it is associated with lens regeneration. The above-mentioned, important regulatory factors may function through these auxiliary factors.

The present study analyzed the spatio-temporal expression of the crystallins during the two processes. The findings indicated that there were significant differences, as well as some similarities between the processes of lens development and lens regeneration, as Henry [23] proposed that the process of the transdifferentiation shares many similarities to that of embryonic lens formation but there are also some interesting differences. Some of differences may be associated with the process of wound healing and cellular dedifferentiation that may be association with lens regeneration [38,39,51]. The data presented here point to crystallins expression, and thus, do not single out a particular mechanism that causes the differences in the two processes. Therefore, further studies are needed to reveal it.

## References

[1] Stocum DL (2002). Regenerative biology and medicine.. J Musculoskelet Neuronal Interact.

[2] Tsonis PA, Madhavan M, Tancous EE, Del Rio-Tsonis K (2004). A newt's eye view of lens regeneration.. Int J Dev Biol.

[3] Tsonis PA, Del Rio-Tsonis K (2004). Lens and retina regeneration: transdifferentiation, stem cells and clinical applications.. Exp Eye Res.

[4] Brockes JP, Kumar A (2002). Plasticity and reprogramming of differentiated cells in amphibian regeneration.. Nat Rev Mol Cell Biol.

[5] Straube WL, Tanaka EM (2006). Reversibility of the differentiated state: regeneration in amphibians.. Artif Organs.

[6] Chaar ZY, Tsilfidis C (2006). Newt opportunities for understanding the dedifferentiation process.. ScientificWorldJournal.

[7] Stone LS (1965). The Regeneration of the Crystalline Lens.. Invest Ophthalmol.

[8] Freeman G (1963). Lens regeneration from the cornea in Xenopus laevis.. J Exp Zool.

[9] Bosco L (1988). Transdifferentiation of ocular tissues in larval Xenopus laevis.. Differentiation.

[10] Filoni S, Bernardini S, Cannata SM (2006). Experimental analysis of lens-forming capacity in Xenopus borealis larvae.. J Exp Zool A Comp Exp Biol.

[11] Henry JJ, Elkins MB (2001). Cornea-lens transdifferentiation in the anuran, Xenopus tropicalis.. Dev Genes Evol.

[12] Gwon AE, Gruber LJ, Mundwiler KE (1990). A histologic study of lens regeneration in aphakic rabbits.. Invest Ophthalmol Vis Sci.

[13] Lois N, Dawson R, McKinnon AD, Forrester JV (2003). A new model of posterior capsule opacification in rodents.. Invest Ophthalmol Vis Sci.

[14] Call MK, Grogg MW, Del Rio-Tsonis K, Tsonis PA (2004). Lens regeneration in mice: implications in cataracts.. Exp Eye Res.

[15] Piatigorsky J (1992). Lens crystallins. Innovation associated with changes in gene regulation.. J Biol Chem.

[16] Altmann CR, Chow RL, Lang RA, Hemmati-Brivanlou A (1997). Lens induction by Pax-6 in Xenopus laevis.. Dev Biol.

[17] McDevitt DS, Brahma SK (1973). Ontogeny and localization of the crystallins during embryonic lens development in Xenopus laevis.. J Exp Zool.

[18] Brunekreef GA, van Genesen ST, Destree OH, Lubsen NH (1997). Extralenticular expression of Xenopus laevis alpha-, beta-, and gamma-crystallin genes.. Invest Ophthalmol Vis Sci.

[19] Brahma SK, McDevitt DS (1974). Ontogeny and localization of the lens crystallins in Xenopus laevis lens regeneration.. J Embryol Exp Morphol.

[20] Campbell JC (1965). An Immuno-Fluorescent Study of Lens Regeneration in Larval Xenopus Laevis.. J Embryol Exp Morphol.

[21] McDevitt DS, Brahma SK (1981). Ontogeny and localization of the alpha, beta, and gamma crystallins in newt eye lens development.. Dev Biol.

[22] Mizuno N, Mochii M, Takahashi TC, Eguchi G, Okada TS (1999). Lens regeneration in Xenopus is not a mere repeat of lens development, with respect to crystallin gene expression.. Differentiation.

[23] Henry JJ (2003). The cellular and molecular bases of vertebrate lens regeneration.. Int Rev Cytol.

[24] Filbin MT (2006). How inflammation promotes regeneration.. Nat Neurosci.

[25] Leon S, Yin Y, Nguyen J, Irwin N, Benowitz LI (2000). Lens injury stimulates axon regeneration in the mature rat optic nerve.. J Neurosci.

[26] Brockes JP, Kumar A, Velloso CP (2001). Regeneration as an evolutionary variable.. J Anat.

[27] Harty M, Neff AW, King MW, Mescher AL (2003). Regeneration or scarring: an immunologic perspective.. Dev Dyn.

[28] Godwin JW, Brockes JP (2006). Regeneration, tissue injury and the immune response.. J Anat.

[29] Brockes JP, Kumar A (2008). Comparative aspects of animal regeneration.. Annu Rev Cell Dev Biol.

[30] Yoshii C, Ueda Y, Okamoto M, Araki M (2007). Neural retinal regeneration in the anuran amphibian Xenopus laevis post-metamorphosis: transdifferentiation of retinal pigmented epithelium regenerates the neural retina.. Dev Biol.

[31] Martinez-Morales JR, Signore M, Acampora D, Simeone A, Bovolenta P (2001). Otx genes are required for tissue specification in the developing eye.. Development.

[32] Köster RW, Kuhnlein RP, Wittbrodt J (2000). Ectopic Sox3 activity elicits sensory placode formation.. Mech Dev.

[33] Chow RL, Lang RA (2001). Early eye development in vertebrates.. Annu Rev Cell Dev Biol.

[34] Ishibashi S, Yasuda K (2001). Distinct roles of maf genes during Xenopus lens development.. Mech Dev.

[35] Ring BZ, Cordes SP, Overbeek PA, Barsh GS (2000). Regulation of mouse lens fiber cell development and differentiation by the Maf gene.. Development.

[36] Cui W, Tomarev SI, Piatigorsky J, Chepelinsky AB, Duncan MK (2004). Mafs, Prox1, and Pax6 can regulate chicken betaB1-crystallin gene expression.. J Biol Chem.

[37] Madhavan M, Haynes TL, Frisch NC, Call MK, Minich CM, Tsonis PA, Del Rio-Tsonis K (2006). The role of Pax-6 in lens regeneration.. Proc Natl Acad Sci USA.

[38] Malloch EL, Perry KJ, Fukui L, Johnson VR, Wever J, Beck CW, King MW, Henry JJ (2009). Gene expression profiles of lens regeneration and development in Xenopus laevis.. Dev Dyn.

[39] Henry JJ, Carinato ME, Schaefer JJ, Wolfe AD, Walter BE, Perry KJ, Elbl TN (2002). Characterizing gene expression during lens formation in Xenopus laevis: evaluating the model for embryonic lens induction.. Dev Dyn.

[40] Mizuno N, Mochii M, Yamamoto TS, Takahashi TC, Eguchi G, Okada TS (1999). Pax-6 and Prox 1 expression during lens regeneration from Cynops iris and Xenopus cornea: evidence for a genetic program common to embryonic lens development.. Differentiation.

[41] Mizuno N, Ueda Y, Kondoh H (2005). Requirement for betaB1-crystallin promoter of Xenopus laevis in embryonic lens development and lens regeneration.. Dev Growth Differ.

[42] Walter BE, Tian Y, Garlisch AK, Carinato ME, Elkins MB, Wolfe AD, Schaefer JJ, Perry KJ, Henry JJ (2004). Molecular profiling: gene expression reveals discrete phases of lens induction and development in Xenopus laevis.. Mol Vis.

[43] Nieuwkoop PD Faber JNormal table of Xenopus laevis (daudin). New York & London: Garland Publishing, Inc199415064684

[44] Schaefer JJ, Oliver G, Henry JJ (1999). Conservation of gene expression during embryonic lens formation and cornea-lens transdifferentiation in Xenopus laevis.. Dev Dyn.

[45] Mizuno N, Agata K, Sawada K, Mochii M, Eguchi G (2002). Expression of crystallin genes in embryonic and regenerating newt lenses.. Dev Growth Differ.

[46] Ingolia TD, Craig EA (1982). Four small Drosophila heat shock proteins are related to each other and to mammalian alpha-crystallin.. Proc Natl Acad Sci USA.

[47] Jakob U, Gaestel M, Engel K, Buchner J (1993). Small heat shock proteins are molecular chaperones.. J Biol Chem.

[48] Horwitz J (1992). Alpha-crystallin can function as a molecular chaperone.. Proc Natl Acad Sci USA.

[49] Chen WV, Fielding Hejtmancik J, Piatigorsky J, Duncan MK (2001). The mouse beta B1-crystallin promoter: strict regulation of lens fiber cell specificity.. Biochim Biophys Acta.

[50] Shekhawat DS, Jangir OP, Prakash A, Pawan S (2001). Lens regeneration in mice under the influence of vitamin A.. J Biosci.

[51] Carinato ME, Walter BE, Henry JJ (2000). Xenopus laevis gelatinase B (Xmmp-9): development, regeneration, and wound healing.. Dev Dyn.

